# Comprehensive Assessment of Druggable Targets in Cortical Neurons Reveals Biological Limits of Cell Type-Specific Neuropharmacology

**DOI:** 10.3390/biomedicines14040823

**Published:** 2026-04-03

**Authors:** Leonie Ripp, Dennis Kätzel

**Affiliations:** Institute of Applied Physiology, Ulm University, 89081 Ulm, Germany

**Keywords:** differential gene expression, single-cell transcriptomics, SmartSeq, CytosploreViewer, mental disorders, drug targets, target discovery, psychiatry

## Abstract

**Background**: Translational circuit neuroscience delivers many candidate neurons whose manipulation could ameliorate psychiatric symptoms. However, the translation of these cellular targets into molecular targets—proteins selectively expressed in those neurons that could be pharmacologically manipulated for treatment—remains scarce. To what extent such a translation is possible or is actually impeded by a lack of highly cell type-specific expression of druggable proteins is unknown. **Methods**: We performed combinatorial differential expression analysis for over 7200 putatively druggable genes (Illuminating the Druggable Genome database) on large-scale single-cell RNAseq datasets from mouse and human cortex (Allen Institute Cell Types Database) to identify selectively expressed genes in important cellular candidates: several pyramidal cell types and parvalbumin, somatostatin and VIP interneurons of the prefrontal and anterior cingulate cortex and hippocampus in mice, and the cingulate cortex in humans. **Results**: We identified dozens of targets, including some with psychiatric relevance and/or suitability to modulate neural activity, like ion channels, GPCRs and transporters. However, none of them were expressed with absolute specificity in any of the analysed target cell types but only stood out in some comparisons, not others. Generally, results depended strongly on selectivity criteria: less conservative approaches (such as moderate *p*-value adjustment or grouping of contrast cell sets) yielded more targets, whereas the introduction of additional plausible constraints (difference in proportion of expressing cells, beta; absence of expression in contrast cell type) drove numbers towards zero. Generally, interneurons showed more selectively expressed targets in comparison to cells of the same region compared to excitatory ones (intra-regional comparisons), whereas the reverse was found in inter-regional contrasts comparing the same cell type across regions. **Conclusions**: The lack of high selectivity in the expression of genes encoding druggable targets constitutes a principal biological limit for manipulating cortical neurons of one type, specifically to leverage therapeutic action. While, currently, this conclusion is limited to the investigated neocortical and hippocampal regions, it highlights the need to develop biological heuristics for identifying targets expressed with relative specificity.

## 1. Introduction

The technological advances in cell type-specific manipulation commonly referred to as “circuit neuroscience techniques” have provided an unprecedented knowledge base on the role of specific types of neurons in psychological functions relevant to psychiatric disorders over the past two decades. Such techniques include optogenetics and chemogenetics [1,2], which allow for the manipulation of specific cell types or even signalling cascades within them in behaving animals. Cell type-specific knockout technology, like Cre-lox or CRISPR/Cas9, in turn, enables the identification of the role of specific proteins for neural function [3,4,5,6].

**Figure 1:**
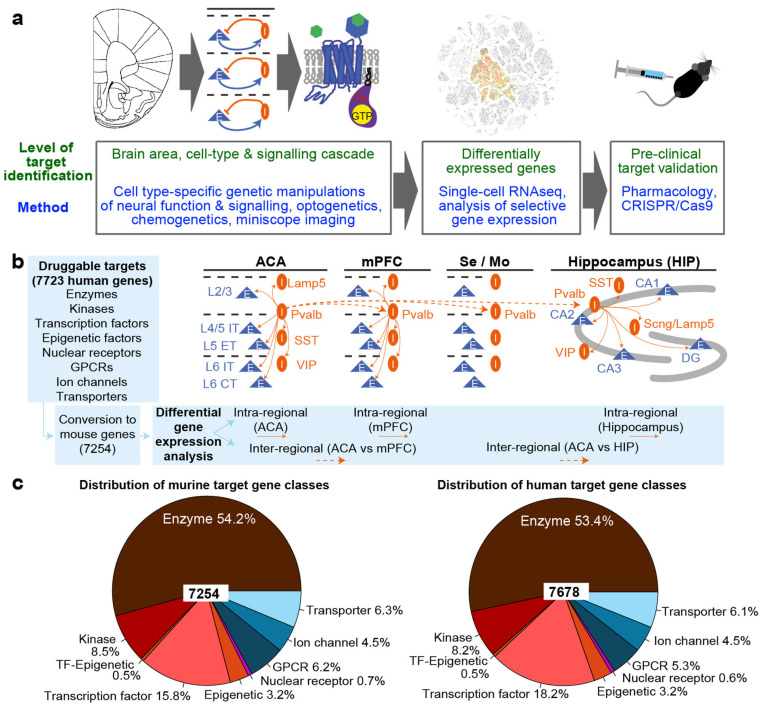
Analysis approach for determining differential gene expression (DGE) between cortical neurons. (**a**) Original motivation for the investigation: circuit neuroscience techniques identify cellular targets to improve symptoms of mental disorders (**left**), which need to be translated into molecular targets by identifying differentially expressed genes in the causally involved cell types (middle) and be validated thereafter in vivo (**right**). Elements reused and adapted from ref. [11]. (**b**) Analysis approach of the current study: likely druggable target genes (from the Illuminating the Druggable Genome Program, IDG) spread over 9 classes (**left**) are converted into their corresponding mouse gene names (**bottom left**) to use them for translational DGE analysis. This is conducted by comparing target gene expression between the major cell classes within murine ACA, mPFC and hippocampus (solid arrows) as well as between cells of the same type that are present in different brain regions (dashed arrows), including also aggregated sensory (Se) and motor (Mo) neocortical areas. This scheme illustrates this for a single cell type, Pvalb interneurons. Orange ovals depict inhibitory cells (I), and blue triangles show excitatory cells (E). (**c**) Distribution of the potentially druggable target genes derived from the IDG list across the 9 IDG target classes, coded by colour, used in this study for the mouse (**left**) and human (**right**) analysis. TF, transcription factor. Note that kinases also include receptor kinases, i.e., receptors, and the ion channel and transporter categories also include genes associated directly with their function, even though they might not directly constitute an ion channel or transporter themselves.

Historically, one of the original promises of this knowledge gain was to provide a renaissance in stalled drug development in psychiatry [1,7,8]. The principal logic of the application of such techniques to fulfil this promise is based on a three-step target discovery process at the pre-clinical level (Figure 1a) [9,10]: (1) The selective and reversible activation or inhibition of neurons using optogenetics or chemogenetics in animals undergoing translationally relevant behavioural tests could reveal which brain areas and which cell types within those areas are causally controlling a certain affective or cognitive function. Using chemogenetics or other techniques in the determined cell types, one could also reveal which signalling cascades in those neurons would be therapeutically beneficial. (2) Once identified, one can use RNA-sequencing or similar techniques to identify selectively expressed genes in such cell types that could activate the same signalling mechanisms. (3) Classical pharmacology, CRISPR/Cas9 or other techniques in behaving animals can serve to validate the identified targets.

We previously followed this approach to identify novel targets to improve the cardinal symptoms of attention-deficit–hyperactivity disorder (ADHD) and identified pyramidal cells of layer 5 and parvalbumin neurons in the anterior cingulate cortical area (ACA) as putative cellular targets, the G_i_- and G_q_-protein cascades, respectively, within those cell types as molecular targets, and mGluR2 as a putative and behaviourally effective drug target expressed with relative specificity in mouse and human ACA layer 5 pyramidal neurons [11,12]. However, maybe the most striking discovery was that among the 400 GPCR target genes encoded in both the mouse and the human genome, none was fully selectively expressed in the two cellular targets of interest [11,12]. For parvalbumin interneurons, we did not find any suitable G_q_PCR expressed with high selectivity, and layer 5 IT pyramidal cells expressed only eight GPCRs significantly higher than other ACA cell types at a level of >3-fold-change. Of these, only three were G_i_-coupled (*Grm2/3/7*), and only one of those (*Grm2*, encoding mGluR2) reached close to a 10-fold higher expression, an appreciable spread in the share of expressing cells (beta > 20%), and a low level of average expression in the undesired (“contrast”) cell types [11]. But even *Grm2* is expressed in other cell types and brain regions that were not included in this analysis. Also, qualitative inspection of expression levels across the 400 GPCR genes suggested that different neuron types do not show much variation with respect to their GPCR expression profiles, i.e., GPCRs expressed in one type of neuron are most likely also expressed in others. This led to our hypothesis that the target discovery pipeline illustrated in Figure 1a is most constrained by a principal biological limit to the selectivity with which GPCRs (or other druggable genes) are expressed in neurons.

Equally, although circuit-neuroscience research in the past two decades has made remarkable advances in delineating the brain regions and cell types that causally influence specific psychological functions (step 1 in Figure 1a) [2,13], the resulting targets, let alone emerging psychiatric therapies, remain scarce.

Therefore, we here follow a top-down approach to delineate to what extent there is a principle biological limit for the identification of druggable targets encoded by genes that are selectively expressed in psychiatrically relevant neuronal cell types. In this analysis, we expand the relevant pharmacological targets from 400 GPCR-encoding genes, to the 7723 genes encoding potentially druggable targets according to the Pharos database (NIH Illuminating the Druggable Genome Program, IDG) [14,15]. Putatively druggable genes are determined by the NIH IDG programme and catalogued in Pharos, if their protein products are considered amenable to pharmacological modulation based on accumulated biological or chemical evidence. This includes membership in established protein families with other targeted members (e.g., GPCRs, kinases, etc.), the presence of known bioactive ligands or even compounds with defined mechanisms of action, or other supporting functional and disease-relevant data. These targets are further stratified into Target Development Levels (TDLs), which reflect the type and strength of supporting evidence. Class-specific quantitative activity thresholds (ligand binding affinities) are specifically relevant for targets supported by chemical evidence (Tchem), whereas other categories rely on clinical or biological data [14]. The IDG list is particularly suitable for this investigation, as it integrates large-scale, multi-modal evidence that is systematically curated and organized in TDLs. It is specifically designed to widen the space of potentially therapeutically modifiable proteins, focusing not only on well-studied but also on under-studied targets, and is based on manually curated entries; scoring criteria for inclusion are standardized and based on a staged system of evidence, integrating a large yet tractable multitude of resources and knowledge base [14,16]. Further, we expand the number of analysed brain regions and cell types to include all major neuron types of the medial prefrontal cortex (mPFC), anterior cingulate cortex (ACA) and hippocamps as targets, and further motor and sensory areas as additional non-targets (herein termed contrast sets). Thereby, we cover neural circuits clearly implicated in major psychiatric disorders: PFC and ACA have both been causally implicated in ADHD [17,18,19], schizophrenia [20,21], autism spectrum disorder (ASD) [22,23] and anxiety and posttraumatic stress disorders [24,25]; the ACA is also central in pain processing [26], impulsive and compulsive behaviour [27]. Equally, the hippocampus, often specific ones of its subfields (dentate gyrus (DG); cornua ammonis areas 1, 2, 3 (CA1/2/3); subiculum (Sub)), has been strongly implicated in schizophrenia [10,28,29,30,31] and depression [32,33] but also in neurological disorders like Alzheimer’s [34,35] and epilepsy [36]. Hence, we selected such regions and key cell types determining information processing within them, as they are likely important cellular targets to treat such diseases. Given the available transcriptomic data, we assessed all three regions in mice and the ACA in humans.

## 2. Materials and Methods

### 2.1. Gene Expression Data

Two large-scale single-cell RNA-sequencing datasets produced and made available by the Allen Institute for Brain Sciences were incorporated in the current study: (1) the “Mouse-Whole Cortex & Hippocampus-Smart Seq” dataset (2019), including 76,307 transcriptomes from all cell types of 20 differentiated forebrain areas, taken from neocortex and hippocampus of ~8-week-old male and female C57BL/6 mice [37]; (2) the “Human Multiple Cortical Areas-SMART-seq” dataset (2019) containing 49,417 single nucleus transcriptomes from six cortical areas: middle temporal gyrus, anterior cingulate cortex, primary visual cortex, primary motor cortex, primary somatosensory cortex and primary auditory cortex, derived from 3 donors. The latter samples were collected from post-mortem, and for MTG also from neurosurgical, donor brains and its expression profiled with SMART-Seq v4 RNA-sequencing [37]. In both datasets, brain region and cortical layer identity were preserved during the dissection process, allowing cells to be labelled accordingly. In addition to their anatomical origin, cell identities had been inferred from gene expression profiles using an iterative clustering pipeline described by Tasic et al. (2018) [38]. The resulting transcriptomic cell types include different subclasses (e.g., L2/3 IT Cxcl14, Pvalb, VIP).

### 2.2. Selection of Brain Regions, Cell Types and Comparisons

**Figure 2:**
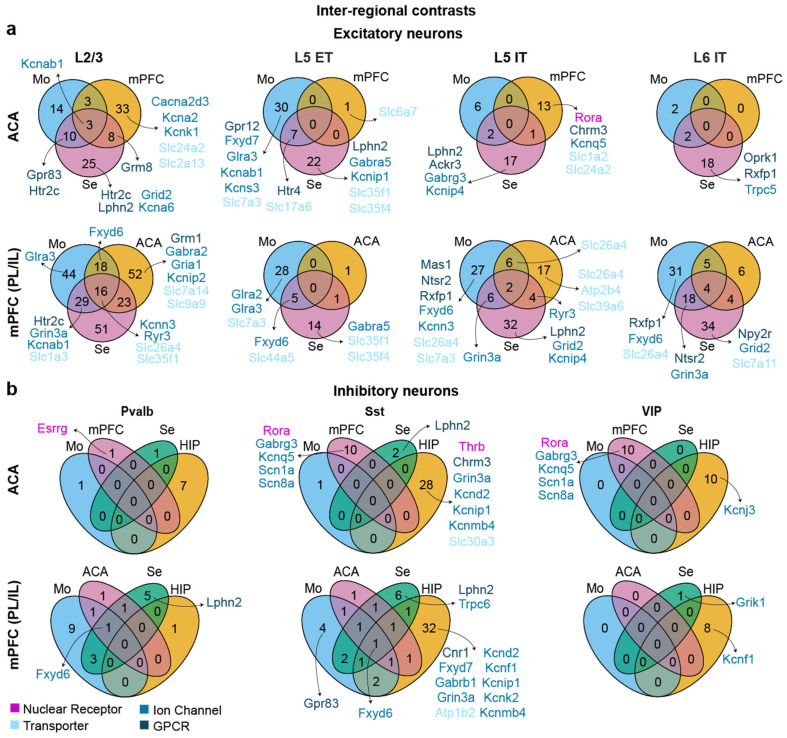
Cell type-specific inter-regional differential gene expression analysis of murine ACA and mPFC. Individual excitatory (**a**) and inhibitory (**b**) neuronal cell types from the ACA (top rows in each panel) and mPFC (bottom rows in each panel) were compared to their corresponding cell types in other cortical regions, including motor cortex (Mo), sensory cortex (Se), hippocampus (HIP) and the respective complementary prefrontal region (mPFC or ACA). Numbers of identified DHETGs in each intersection of comparisons are stated, and the subgroups of translationally more relevant targets (nuclear receptors, transporters, ion channels and GPCRs), coded by colour, are stated next to the respective intersection area of each Venn diagram.

Brain areas for in-depth analysis were selected based on the availability of single-cell data and documented psychiatric relevance (see Introduction). For mice, we analysed all three intended areas (hippocampus, ACA, and mPFC comprising prelimbic and infralimbic cortex), while for the human dataset, only the ACA-homologue cingulate cortex (CgC) fulfilled this criterion, given the lack of human PFC or hippocampus data in the used Allen Institute database, usable for a comparative analysis. The remaining brain regions mostly comprised primary or secondary motor (Mo) or sensory (Se) areas in both species, which were included as contrast regions (i.e., regions where expression of a target in the equivalent cell type should be low; Figure 2b).

As for the brain areas, cell types were selected based on documented or presumed relevance for psychiatric disorders or cortical information processing in general. Using the metadata of the respective datasets, lists of individual cells (using the cells ID in the column “sample_name”) were compiled by filtering the dataset first by “region_label” to select samples from the desired brain area, then by “subclass_label” to isolate the specific interneuron or excitatory subtype. For analysis comparing all GABAergic or all glutamatergic cells, the column “class_label”, which designates each sample as “glutamatergic”, “GABAergic”, or “non-neuronal”, was used. For murine ACA and mPFC, the used cell types are stated in Table 1.

For analysis of cell types of the hippocampus, at first, samples annotated with the region labels “HIP,” “CA,” and “SUB;ProS” were selected, and within that regional subset, the five principal excitatory projection-neuron types of CA1, CA2, CA3, dentate gyrus (DG), and subiculum (SUB), as well as the three interneuron subclasses Sst, VIP, and Pvalb, were retained (Table 2).

For human ACA analysis, only the major interneuron types Pvalb, Sst and VIP were analysed as target cells, because the pyramidal cell classes in the dataset are not clearly delineated or separable by layer as many subgroups, especially the inter-telencephalic (IT) groups, contain cells from multiple layers. Therefore, five excitatory groups were formed but only used as contrast sets in addition to two additional interneuron groups (PAX6, LAMP5; Table 3).

For each of those areas, two differential gene expression (DGE) analyses were performed: (1) comparing different cell types within each area, and (2) comparing the same cell types between distinct cortical brain regions (including not only the stated areas but also all areas that qualify as sensory or motor cortex, for which data were available) in case the respective cell types were present in both areas (Figure 2b). All classes, subclasses and individual cells used in this study are presented in Supplementary Files S1 and S2.

### 2.3. Selection of Target Genes

To identify potential drug targets, we resorted to the, to our knowledge, most comprehensive list of genes encoding potentially druggable proteins in the human body, which is the Pharos database of the NIH Illuminating the Druggable Genome (IDG) Program (2023; https://pharos.nih.gov/, accessed on 2 February 2026). It lists 7723 druggable targets (in 2023) based on databases, experiments and the literature. It distinguishes them into the nine following non-overlapping classes: GPCR, ion channel, transporter, kinase, enzyme, nuclear receptor, transcription factor, transcription factor-epigenetic, epigenetic.

Since this database lists human genes only, we used the 110 version of the Ensembl genome browser (Ensembl Project) and subsequent manual inspection to identify the equivalent murine gene names for the mouse gene expression analysis. In the Ensembl genome browser, the option “BioMart” and then the “Ensembl Genes 110” database and the “HumanGenes” dataset were selected, to convert the IDG list of human gene names to a list of the corresponding mouse gene orthologues. Human gene names that lacked a murine orthologue were ignored. For some human genes, BioMart returned multiple murine orthologues, each of which was added separately to the mouse target list. Conversely, duplicate entries of the same mouse gene resulting from mappings to multiple human genes were removed. This led to a temporary target list containing 7271 mouse genes. Subsequently, this list of mouse target genes was aligned with the list of the 45,768 genes of the “Mouse-Whole Cortex & Hippocampus-Smart Seq” dataset, as listed in the output of Cytosplore Viewer (Erasmus MC Cytosplore Team, 2025; https://viewer.cytosplore.org/ (accessed on 21 September 2023)) [37]. This revealed that 245 IDG target genes were not present in the dataset. Gene name aliases of these targets were determined by manual searches in the NCBI Gene database (National Center for Biotechnology Information (NCBI), 2023), filtering for “house mouse/Mus musculus”. While 228 of the non-matching target names could be identified in the dataset and were replaced by their aliases, no matches could be found for 17 genes, which were removed from the target list. As a result of this multi-step alignment process, 7254 gene names remained on the murine target list, across the nine IDG classes (Figure 1c).

An identical alias resolution procedure was applied to the human target list using the metadata from the “Human Multiple Cortical Areas–SMART-seq” dataset, which contained 50,281 genes. At first, 13 duplicate entries were removed from the IDG target list. Of the remaining genes, 180 did not match any entries in the dataset. For 148 of these, gene name aliases were identified manually using the NCBI Gene database, which were then added to the target list. The remaining 30 could not be resolved and were removed. This resulted in a final human target list comprising 7678 genes, again spanning all nine IDG families (Figure 1c). All human and mouse target genes, including their IDG class, are presented in Supplementary File S3.

### 2.4. Contrasts for Comparison of Gene Expression Between Cell Types Within One Area

Differential gene expression (DGE) between different cell types of a single brain region was calculated between each *target* cell type and every one of the other cell types (*contrast* set) one by one, and then the remainder of the intersection of all pairwise comparisons involving the same target cell type constituted the list of differentially higher expressed target genes (DHETGs). This conservative or one-vs-one approach was used for all 4 target regions analysed here (mouse mPFC, ACA and hippocampus; human ACA). Note that, in each case, the number of contrast cells exceeded those of the target cells; additional contrast cell types (that were not used as target cell types due to a lack of psychiatric implication in the mouse dataset or overlapping delineation in the human dataset) were L6b pyramidal cells in murine mPFC and ACA, Lamp5 and Sncg interneurons in hippocampus, and 7 sets of excitatory or inhibitory cells (L5 ET, L5/6NP, L6CT, L6b, IT, Pax6, Lamp5) for human ACA (see Section 2.2 and Table 1 and Table 2). When comparing SUB to CA1p and vice versa for the hippocampus dataset, the combined subclass CA1sp SUBsp Kcnip1 was omitted from both target and contrast sets to avoid comparing very similar cell subtypes in the analysis.

**Figure 3:**
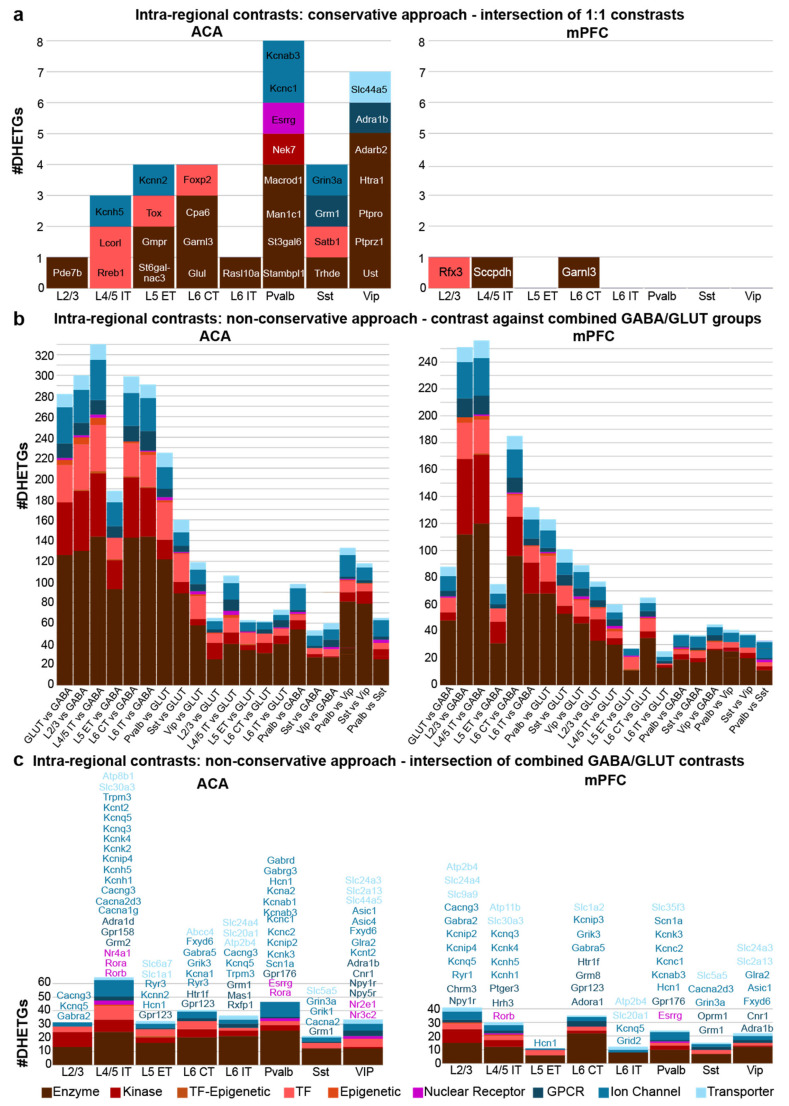
Intra-regional differential gene expression analysis identifying differentially higher expressed genes (DHETGs) in murine ACA (**left**) and mPFC (**right**). All plots show the number of identified DHETGs for each cell class (**a**,**c**) or individual comparison (**b**), as well as the identity of the targets, colour coded by target class, for either all classes (**a**) or only the four most targetable classes for modulating neural activity (**c**). Intra-regional contrasts were performed using two complementary strategies: (**a**) Results of the conservative approach based on pairwise comparisons between each listed neuronal cell type, as well as L6b neurons (used exclusively as contrast set; 8 comparisons in total the results of which were intersected; see Table 1). (**b**,**c**) Results of a non-conservative approach contrasting each cell type against all GABAergic neurons (GABA) or all glutamatergic neurons (GLUT) pooled, respectively (**b**), and then intersecting the results of those two comparisons for each cell type (**c**). (**b**) also shows additional biologically relevant pairwise comparisons.

In addition to this standard approach, a less stringent (termed non-conservative or one-vs-all) approach was additionally applied for murine PFC and ACA and human ACA, for comparison, whereby DGE was calculated between each target cell type and all other cells belonging to each of the two top classes (GABAergic or glutamatergic, removing the target type) combined, and then the intersection of these two comparisons was taken as the DHETG result. In this approach, multiple other biologically relevant 1:1 comparisons were also conducted (including GABAergic vs. glutamatergic, Pvalb vs. VIP, etc.; see Figure 3b).

### 2.5. Contrasts for Comparison of Gene Expression Between Areas for Each Cell Type

To investigate if it is possible to differentially target cell types in just one brain region, we performed DGE analysis between a given target cell type in one neocortical area and its counterpart in different regions. Cells in murine ACA and mPFC were compared to their counterparts in mPFC and ACA, respectively, and in sensory (Se) and motor areas (Mo). The Se group combined cells from the primary visual cortex (VISp), posterolateral visual area and postrhinal area (VISpI;VISpor), anterolateral visual area and lateral visual area and laterointermediate area (VISAI;VISI;VISli), anteromedial visual area and posteromedial visual area (VISam;VISpm), auditory areas (AUD), primary somatosensory area (SSp), supplemental somatosensory area (SSs), supplemental somatosensory area and gustatory areas (SSs;GU), supplemental somatosensory area and gustatory areas and visceral areas (SSs;GU;VISC) as well as gustatory, visceral and posterior subdivision areas of the agranular insular cortex (GU;VISC;AIp). The Mo groups combined the primary motor cortex (MOp) and secondary motor cortex (MOs). Additionally, the main interneuron subtypes (Pvalb, Sst, VIP) were also compared against their counterparts in the hippocampus. Cells in human ACA were compared to sensory areas (Se), motor areas (Mo) and the medial temporal gyrus (MTG). The Mo group combined the primary motor cortex representing the lower limb (M1lm) and upper limb (M1ul), while the Se group included the primary somatosensory cortex lower (S1lm) and upper region (S1ul), as well as the primary visual (V1C) and auditory cortex (A1C).

In mice, such comparisons were made for both glutamatergic (L2–3, L5 IT, L5 ET, L6 IT) and GABAergic cell types (Pvalb, Sst, VIP), as they have matching categories of neurons, whereas comparisons with the hippocampus could only be conducted for inhibitory neurons, since the excitatory neurons of this region have no obvious counterpart in the glutamatergic neurons of the neocortex. For each cell type, a “one-vs-one” comparison was made with its counterpart in every other regional group (three or four contrasts, depending on the group), and the resulting intersection from those pairwise comparisons constituted the list of DHETGs. In the human brain, only the three major interneuron target classes (Pvalb, Sst, VIP) were analysed, as the pyramidal neurons were not suited for this analysis due to the mentioned difficulty of establishing functionally relevant subtypes.

The conservative approach of regarding a gene as a DHETG only when it stands out in all intersections was chosen because it best represents the analysis goal of the selectivity of expression in only one cell type. However, given the low number of resulting hits, we subsequently used less conservative approaches, as described in the Results.

### 2.6. Calculation of Differential Gene Expression

DGE for the list of cells of each target type and one contrast type at a time (see Supplementary Files S1 and S2) was calculated in CytosploreViewer [37]. The following parameters were extracted from the DGE results (as described previously [11]):

(1) log_2_(1 + CPM(exons + introns)), used to determine the average expression of a gene (GE) in the target set (Set T) and the contrast set (Set C). Here, counts per million (CPM) represent read counts normalised for sequencing depth per cell. The value 1 is added so that genes with zero CPM yield an expression value of 0 after log-transformation. Since the Allen Institute data are based on single-nucleus RNA-seq, a large fraction of transcripts is still unspliced and contains introns. Including both exonic and intronic reads ensures that ongoing transcription is captured.
(2) log_2_(fold-change) = log_2_(GE_Set T_/GE_Set C_) = log_2_(GE_Set T_) − log_2_(GE_Set C_), as primary measure of differential gene expression (Diff_Mean).
(3) *p*-value assessing the significance of the difference in gene expression between the two sets according to the Wilcoxon test, corrected for the number of comparisons using the Bonferroni correction, as is the standard output of CytosploreViewer.

### 2.7. Analysis and Evaluation of Results for Druggable Targets

A customised Igor Pro 6 (WaveMetrics Inc., Lake Oswego, OR, USA, 2025) script [11] was used to extract the resulting DGE parameters stated above for each of the 7254 murine or the 7678 human target genes, respectively, from our target lists. A DHETG was defined according to the following criteria: (1) expression level between the two compared cell types should be significantly different (Bonferroni-corrected *p* < 0.05), and (2) the quantitative difference in expression level (Diff_Mean) should be positive and correspond to an at least *three-fold* higher depth-normalised read count (GE_Set-T_/GE_Set-C_ > +3; i.e., log2(fold-change) > 1.585). As a positive control of our approach, we confirmed that it retrieves established markers (Pvalb, Sst, Vip, Gad1, vGlut1) of the relevant cell classes (Supplementary Table S1).

DHETG numbers and, where graphically possible, identity are rendered in bar graphs and Venn diagrams created in RStudio (RStudio IDE, 2025) using a custom-written R script (R Core Team, 2025), applying colour coding for target class and region or cell type where appropriate. Given the spatial constraints with figure space, DHETG names were stated only for those belonging to the target classes which are most likely useful to alter neural activity, namely nuclear receptor, GPCR, ion channel, and transporter.

## 3. Results

### 3.1. Limited Inter-Regional Differential Expression of Genes Across ACA and mPFC Cell Types

The fundamental logic of identifying distinct cortical cell types based on gene expression [37] suggests that the cortical microcircuit is built from virtually identical cell types irrespective of the area or its function. Therefore, we hypothesized that the similarity of transcriptomes of analogous cell types in functionally distinct brain regions would pose the most significant challenge to identify pharmacological targets that are suitable to selectively modulate a psychiatrically relevant brain region. To test this hypothesis, we performed pairwise comparisons between either ACA or mPFC and the corresponding populations in other regions, including motor cortex, sensory cortex and hippocampus, separately for each potentially psychiatrically relevant cell type. The sets of differentially higher expressed target genes (DHETGs) from each pairwise comparison were intersected between such comparisons to identify those consistently upregulated within a given cell type in the brain region of interest (mPFC or ACA) compared to every other region (Mo, Se, ACA/mPFC).

The analysis showed a profound lack of targets that were highly regionally specific (Figure 2a,b). Even in this limited comparison that only included neocortical areas, the majority of excitatory neurons had either none (L5 ET in both regions, ACA L5 IT and L6 IT) or less than five (ACA L2/3, mPFC L5 IT and L6 IT) DHETGs resulting from the intersection of the three inter-regional comparisons. Only mPFC L2/3 cells featured 16. In general, L2/3 cells of both regions appeared to show more DHETGs, also when investigating intersections of just two regions or 1:1 comparisons, than the other major excitatory neurons of the prefrontal and cingulate cortex, making them a more likely target for pharmacological interventions (Figure 2a). However, such quantitative conclusions need to be drawn with caution, given the very low absolute numbers of identified DHETGs in all cell types, including L2/3 principal cells.

This conclusion holds, when incorporating the three main inhibitory interneuron types of mPFC and ACA in the analysis, all of which showed either only one or no DHETG in the intersections of the comparisons to the other three neocortical areas alone, even before including the hippocampal comparison (Figure 2b). When regarding DHETGs resulting from intersecting only two comparisons and from 1:1 comparisons, it was obvious that Pvalb and VIP appeared to be the most stereotypic neocortical cells, with hardly any DHETGs in inter-regional comparisons, whereas Sst interneurons featured some regional specificity. Secondly, excitatory neurons were generally more regionally specific than inhibitory ones, and mPFC more than ACA. Interestingly, a few DHETGs were shared by different cell types of mPFC, most prominently the regulator of the Na-K-ATPase (and, hence, excitability) *Fxyd6*, over-expressed in Pvalb, Sst and also L5 ET neurons compared to sensory and motor areas, and the adhesion GPCR *Lphn2* (Pvalb, Sst, L5 IT). Generally, despite the lack of high regional selectivity, there were surprisingly many DHETGs encoding genes that have clearly been implicated in psychiatric disorders before (see Section 4.3).

### 3.2. Limited Selective Target Expression in Cell Types Within ACA and mPFC

In t-SNE plots of the complete multi-regional dataset, cells cluster by cell type rather than by brain region [37], suggesting that the genetic difference is larger between different cell types within a region than between the similar cell types across regions. Therefore, we calculated the former. The intersection of the eight 1:1 comparisons was calculated for each one of the major cell types in mPFC and ACA.

In contrast to the hypothesis, this conservative approach yielded a very limited number of DHETGs, ranging between 1 and 8 in ACA and between 0 and 1 in mPFC (Figure 3a). The majority of hits were enzymes; only few were transcription factors or ion channels (five each in ACA) or GPCRs (two in ACA). Opposite to the inter-regional comparison, more DHETGs were found in ACA than in mPFC, and more in ACA interneurons than in excitatory cells, suggesting that interneurons are more distinct to other cell types within the same region but more similar within the same cell types across regions.

Given the realization from both comparisons that absolute selectivity in target expression is an unrealistic goal, we relaxed the intra-regional analysis by calculating DGE in comparison to groups of cell types combined, i.e., against all other glutamatergic or GABAergic cells, at a time (Figure 3b), and then extracted the intersection of both comparisons (“one cell type vs. all others combined”; Figure 3c). We also contrasted the main interneuron types against one another (Figure 3b). This approach revealed many dozens of druggable target genes, which are expressed with moderate specificity in each of the analysed cell types. Even though enzymes were still the largest target group, every cell type also expressed several ion channel-related genes and often even GPCR or transporter genes, which would be expected to allow a more direct modulation of neural activity, particularly L4/5 IT, Pvalb and VIP neurons of both regions, as well as in mPFC L2/3 neurons.

In the initial step (Figure 3b), differentiating excitatory against inhibitory cell types yielded the highest number of DHETGs, whereas the reverse comparison resulted in fewer. Expectedly, comparisons within excitatory or within inhibitory populations yielded the lowest number (although Pvalb and Sst were still remarkably distinct from VIP interneurons). Further, the number of DHETGs found in each comparison was mostly lower in mPFC than in ACA. The intersection (Figure 3c) showed no more obvious differences in the numbers of DHETGs in excitatory and inhibitory neurons.

### 3.3. Intersection of Intra- and Inter-Regional DHETG Sets Reveals Virtual Lack of Selectively Expressed Druggable Targets

**Figure 4:**
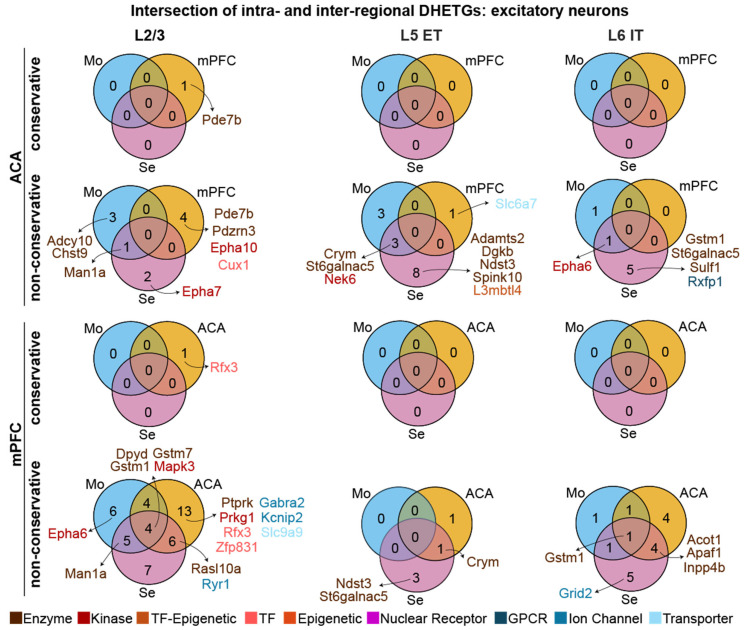
Numbers of identified DHETGs in each intersection of intra- and inter-regional DHETG results (Figure 2 and Figure 3) for ACA (**top**) and mPFC (**bottom**) for four classes of excitatory neurons (stated at the top). Conservative (top rows within each region) and non-conservative intra-regional analyses were intersected with the corresponding inter-regional analyses against motor cortex (Mo), sensory cortex (Se), and the respective complementary prefrontal region (mPFC or ACA) for each cell type. Identified DHETGs are listed next to each Venn diagram and colour coded by gene class.

**Figure 5:**
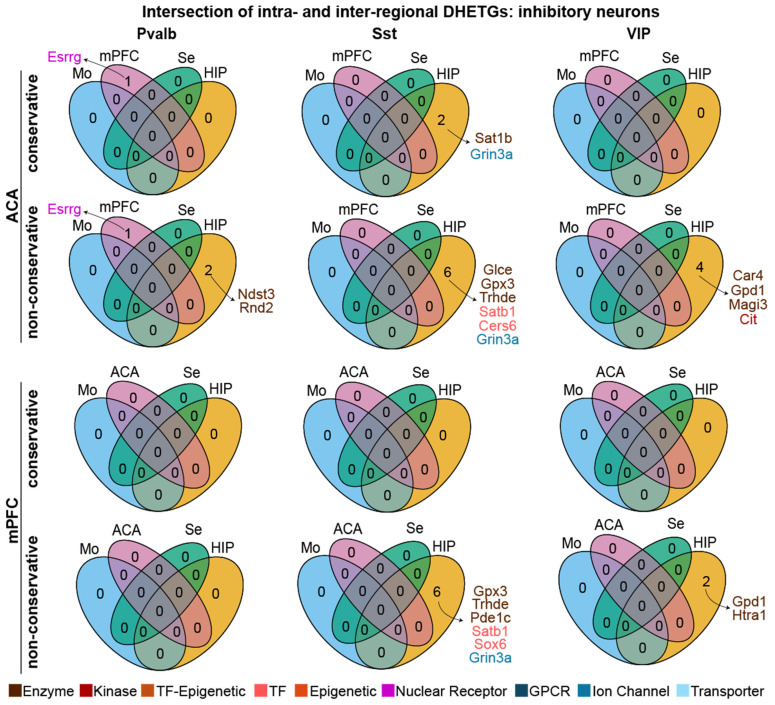
Numbers of identified DHETGs in each intersection of intra- and inter-regional DHETG results (Figure 2 and Figure 3) for ACA (**top**) and mPFC (**bottom**) for three classes of inhibitory neurons (stated at the top). Conservative (top rows within each region) and non-conservative intra-regional analyses were intersected with the corresponding inter-regional analyses against motor cortex (Mo), sensory cortex (Se), and the respective complementary prefrontal region (mPFC or ACA) for each cell type. Identified DHETGs are listed next to each Venn diagram and colour coded by gene class.

Given that both the inter-regional and the intra-regional DGE analyses revealed at least some selectively expressed targets, we next analysed to what extent these identified DHETGs were actually overlapping, which would reassure us of high selectivity. To investigate this, we extracted the intersection of the result from both comparisons for the six cell types, which were identical in both comparisons, the excitatory classes L2/3, L5 ET and L6 IT (Figure 4) and the main inhibitory cell types Pvalb, Sst and VIP (Figure 5).

When intersecting the result from the conservative analysis (top row in each regional subpanel of Figure 4 and Figure 5), virtually no DHETGs were left for any of the six cell types, indicating that genes which showed some selective expression in a given cell type in the intra-regional comparison were distinct from those detected in the inter-regional comparison for the same cell type; hence, none of the few identified targets had absolute specificity. However, when using the DGE results from the less conservative one-vs-all comparisons, and also when looking at intersections with only one or two regional comparisons, several targets did actually emerge for excitatory neurons, especially for mPFC L2/3 cells (Figure 4). This was not the case, however, with inhibitory interneurons: apart from few DHETGs emerging for Sst and VIP neurons when regarding the intersection of the intra-regional comparisons and the mPFC-hippocampus contrast, the number of selectively expressed genes in both comparisons was consistently zero, likely due to the very low inter-regional genetic variability in inhibitory interneurons (Figure 2b).

### 3.4. Limited Number of Selectively Expressed Genes in Intra-Hippocampal Contrasts

**Figure 6:**
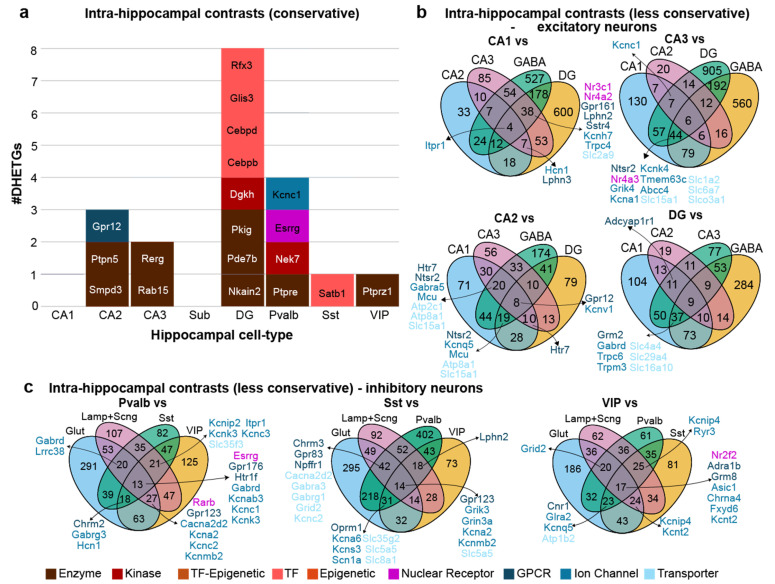
Intra-regional differential gene expression analysis of murine hippocampal neuronal cell types. (**a**) Numbers of DHETGs resulting from conservative analysis based on the intersection of 8 pairwise comparisons between each listed neuronal cell type and all other hippocampal cell types (including pooled Lamp5 + Sncg neurons) used as the contrast set. (**b**) Numbers of DHETGs resulting from less conservative intra-regional analysis of excitatory hippocampal neurons, comparing each excitatory cell type pairwise to other excitatory neurons (except the subgroup) and against all GABAergic neurons combined (4 comparisons). (**c**) Same as (**b**) but for inhibitory hippocampal neurons, comparing each inhibitory cell type pairwise to each of the other inhibitory neurons and against all glutamatergic neurons combined. DHETGs from all (**a**) or the most relevant (**b**,**c**) classes are stated in each to each diagram and colour coded by functional gene class.

Aside from prefrontal and anterior cingulate cortex, the hippocampus is a cortical structure with very high relevance for mental disorders, especially schizophrenia, depression, epilepsy and Alzheimer’s disease. Often, distinct pathologies have been associated with the different subfields (DG, CA1, CA2, CA3) and cell types (including Sst and Pvalb interneurons) [10,31], making it desirable to target excitatory cells of the different subfields or distinct types of inhibitory neurons selectively. To assess the possibility of this pharmacological targeting, we repeated the DGE analysis across those main hippocampal cell type, intersecting DHETG lists from all eight 1:1 comparisons (conservative approach; Figure 6a). Again, target genes expressed with high selectivity were scarce, absent in CA1 and Sub cells, ranging between 1 and 8 in the remaining six cell types. Again, most DHETGs were enzymes or transcription factors, and only one target each belonged to the ion channel or GPCR family, respectively (*Kcnc1* for Pvalb and *Gpr12* for CA2).

Given this scarcity, we relaxed the intersection approach to identify targets with relative specificity, focusing on the most relevant contrast cell types: we grouped all GABAergic cells into a single contrast set (and removed Subiculum neurons) for analysis of excitatory neurons and all glutamatergic cells into one contrast group for the analysis of inhibitory neurons (similar to the non-conservative approach in the neocortex). In this way, every excitatory cell type had 4–9 DHETGs in the complete intersection, and many more, including several ion channels, transporters and GPCRs, in the partial intersections (Figure 6b). As seen in the neocortex, even more targets were found for the three main types of interneurons (13–17 DHETGs for the complete intersection), again featuring several ion channels, GPCRs and transporters (Figure 6c).

### 3.5. Limited Inter- and Intra-Regional Differential Expression of Target Genes in Human ACA

**Figure 7:**
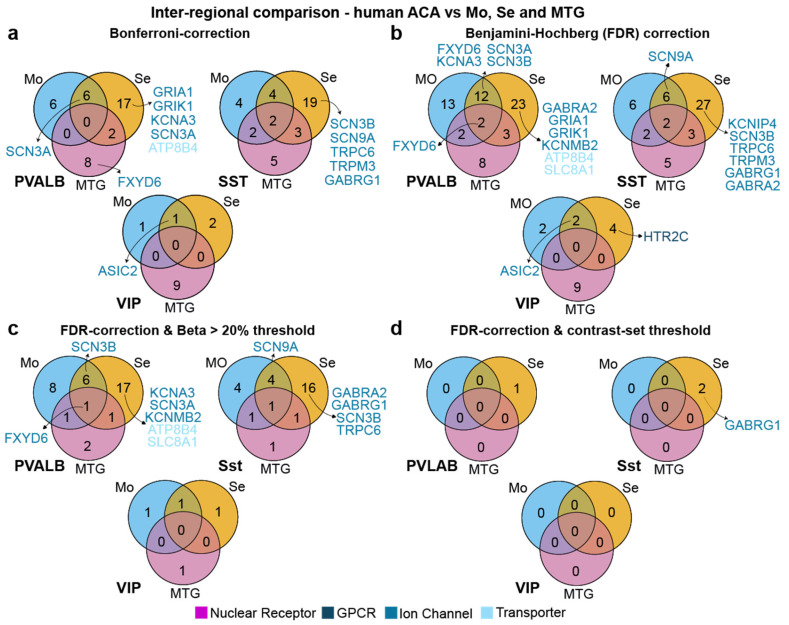
Differential gene expression analysis of inhibitory cell types in the human anterior cingulate cortex (ACA) in comparison to other brain regions. (**a**) Numbers of DHETGs resulting from intersection of 3 pairwise inter-regional comparisons to the corresponding cell types in motor cortex (Mo), sensory cortex (Se), and medial temporal gyrus (MTG) for each of the major ACA interneuron classes (Pvalb, Sst and VIP), using a Bonferroni-corrected *p*-value. (**b**) Same as (**a**), but with Benjamini–Hochberg false-discovery rate (FDR) correction. (**c**) Using the same analysis as (**b**) but applying an additional criterion of beta > 20%. (**d**) Using the same analysis as (**b**) but applying an additional criterion of log_2_(GE_Set-C_) < 1, i.e., requiring absence of expression in the contrast cell type. DHETGs from the most relevant classes are annotated adjacent to each diagram and colour coded by functional gene class.

Although the cell types in the human and rodent neocortex are very much aligned [37], specialisations of neurons and brain areas could have occurred across evolution that increase genetic differences. To assess this possibility in an exemplary manner, we analysed DGE of the translationally most relevant inhibitory cell types of the target region ACA, which can be clearly identified and compared across regions (in contrast to excitatory cell classes in the Human Brain SmartSeq dataset). At first, we extracted DHETGs of these cells in comparison to their homologues in sensory, motor and temporal cortical areas (Figure 7a, Supplementary File S7). Surprisingly, as in mouse ACA, there were hardly any that were significantly higher expressed in ACA interneurons compared to the homologues in the other regions combined: zero for Pvalb and VIP interneurons, and two for Sst interneurons, the enzymes GDA and NKAIN2, out of the 7678 analysed genes. A few targets, including several ion channels, emerged when comparing against only one or two areas at a time, with most differences emerging in comparison to the sensory cortex. Given this low number, we investigated if a more relaxed, yet justifiable, correction for multiple comparisons would lead to more hits, replacing the standard Bonferroni correction (used by CytosploreViewer) with a Benjamini–Hochberg false-discovery rate (FDR) correction. Although this approach increased the number of DHETGs found with at least two intersections, the difference was not substantial (Pvalb cells: 8 → 18; Sst cells: 11 → 13 VIP cells: 1 → 2; Figure 7b). Given the resulting larger number, we next investigated to what extent the introduction of further, biologically plausible constraints on the definition of a DHETG altered this result [11]: Firstly, we added the standard criterion, that the number of expressing target cells should be at least 20% higher than the number of expressing cells in the contrast set (beta > 20%). This substantially decreased the number of DHETGs again by about half in every cell type (Figure 7c). Secondly, we added the criterion that the contrast cells should not express the gene in appreciable amounts, at all, using a standard value of log_2_(GE_Set-C_) < 1; this reduced the number of DHETGs to 0 for all dual or triple intersections in all cell types (Figure 7d). This confirms the notion that interneurons of the same type residing in different neocortical regions cannot be discriminated by DGE.

**Figure 8:**
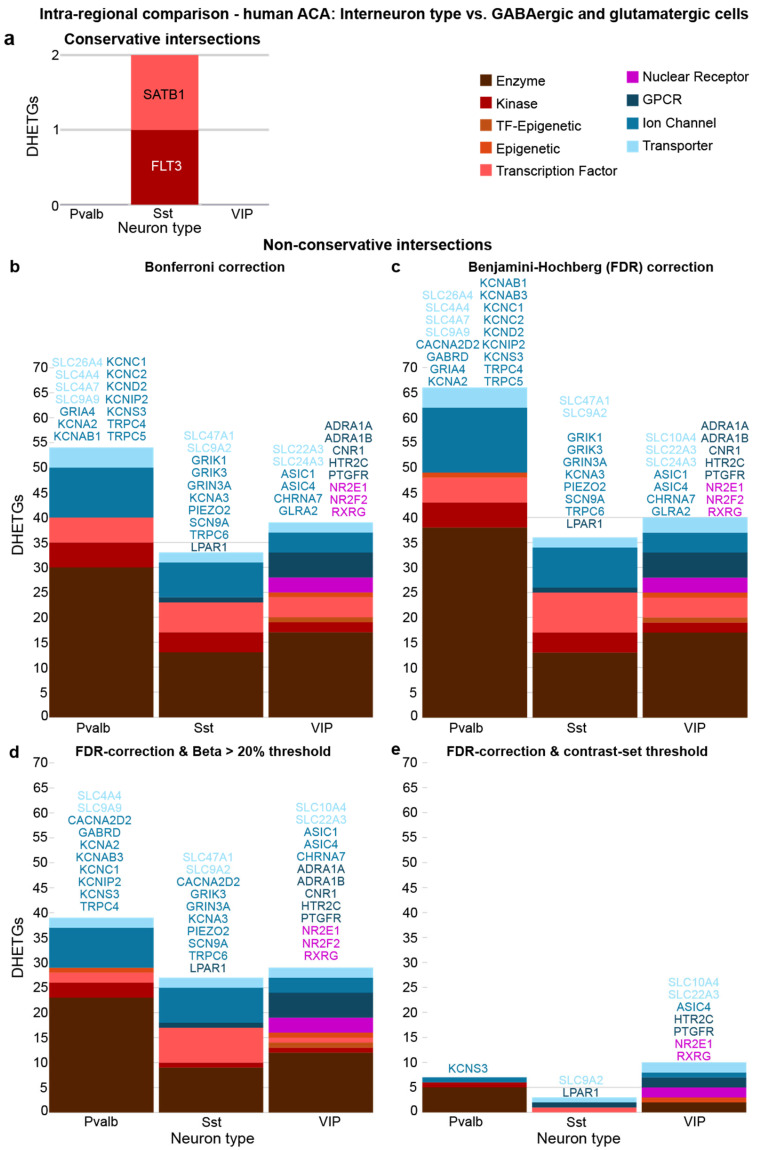
Intra-regional differential gene expression analysis of inhibitory cell types in the human ACA. (**a**) Numbers of DHETGs resulting from intersection of 9 comparisons within ACA for each of the interneuron classes against 4 other interneuron classes (adding PAX6 and LAMP5) and 5 glutamatergic populations (L5 ET, L5/6 NP, L6 CT, L6b, IT). (**b**) Results of a non-conservative approach contrasting each cell type against all GABAergic neurons or all glutamatergic neurons pooled, respectively, and then intersecting the results of those two comparisons for each cell type. (**c**) Same as (**b**), but with Benjamini–Hochberg false-discovery rate (FDR) correction instead of CytosploreViewer’s Bonferroni correction. (**d**) Using the same analysis as (**c**) but applying an additional criterion of beta > 20%. (**e**) Using the same analysis as (**c**) but applying an additional criterion of log_2_(GE_Set-C_) < 1, i.e., requiring absence of expression in the contrast cell type. DHETGs from the most relevant classes are annotated adjacent to each diagram and colour coded by functional gene class.

Secondly, again expecting more results, we compared each of the three interneuron types against not only the remaining two but also the other neuron types in ACA grouped into seven subclasses (see Section 2.2). When intersecting the results from these nine comparisons, neither Pvalb nor VIP interneurons showed any remaining DHETGs, and Sst interneurons showed only two (Figure 8a), which were distinct from the targets emerging from inter-regional intersections (Figure 7a). This suggests that our conclusion from the analysis of the mouse dataset also holds true (in fact, even more) for the human cortex. Cellular targets show hardly any selectively expressed druggable targets.

Given this scarcity, we relaxed the intersectional approach to a non-conservative one, as done for the mouse ACA data before (Figure 3c; Supplementary File S8), emphasizing the absence of expression in the cells from the combined main classes (GABAergic or glutamatergic). This yielded substantially more DHETGs (33–54, depending on interneuron type; Figure 8b), including many ion channel- and transporter-related genes in Pvalb and Sst neurons and several GPCRs in VIP neurons. We then applied the same relaxation of *p*-value correction (FDR instead of Bonferroni correction; Figure 8c), and the introduction of an additional expression difference (beta > 20%, Figure 8d) or contrast set expression criterion (log_2_(GE_Set-C_) < 1; Figure 8e), as done with the inter-regional analysis, before.

FDR correction increased the number of DHETGs mildly and mainly in Pvalb neurons (Figure 8c), whereas the introduction of the beta threshold decreased numbers by about 1/3 in each neuron type, still leaving a substantial number of interesting targets. This suggest that the inclusion of a beta threshold is a reasonable measure to constrain the target list towards more selective expression, without being overly conservative. Adding the requirement of absent expression in the target set reduced the number of DHETGs drastically, but, in contrast to the inter-regional analysis, not to zero. VIP cells still displayed 10 DHETGs, of which 7 belonged to the classes that are likely most suitable to modulate neural activity. Notably, this short list also contained the serotonin-receptor gene HTR2C, which also appeared in the inter-regional comparison against VIP cells in the somatosensory cortex (Figure 7b and Figure 8e). Overall, this comparative view on five biologically and statistically plausible strategies for determining DHETGs suggests that a combination of criteria may be better suited to yield relevant targets than a conservative, fully intersectional approach with strict *p*-value correction.

### 3.6. The Number of Selectively Expressed Target Genes Differs by Protein Class

**Figure 9:**
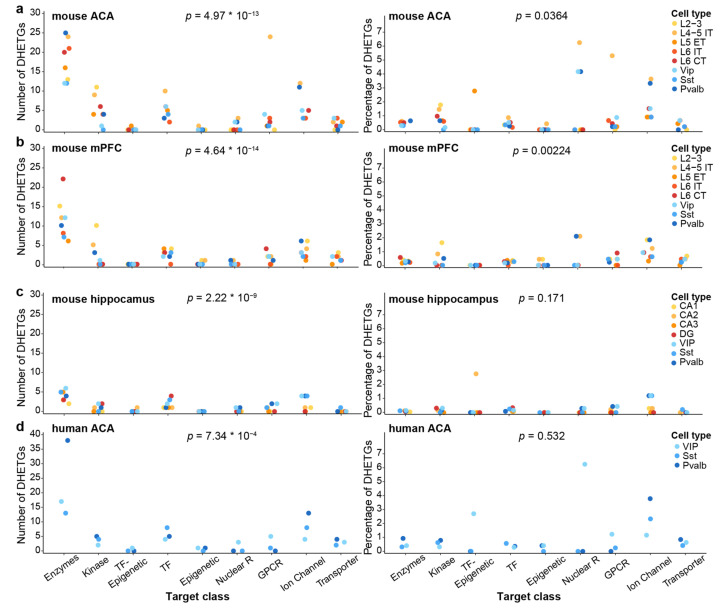
Absolute and relative number of DHETGs identified in less conservative intra-regional analyses aggregated by target class. (**a**) Mouse ACA. (**b**) Mouse mPFC. (**c**) Mouse hippocampus. (**d**) Human ACA. Cell types are coded by colour (indicated on the right). The percentage was calculated relative to the total number of genes in each target class (plotted in %). *p*-values at the top of each subpanel were derived from one-way ANOVA across target class, treating cell types as samples.

Distinct classes of genes not only differ by their functional role in shaping neuronal excitability but, because of that, may also differ in the number of their members that are differentially expressed in neurons. To assess this systematically and representatively, we aggregated the number of DHETGs for each target cell type across all non-conservative intra-regional comparisons in the three investigated mouse regions and human ACA (Figure 9). As is already evident from Figure 3, Figure 6a and Figure 8, in absolute numbers, enzymes and, to a lesser extent, kinases and ion channels were more common among the DHETGs compared to the other classes (Figure 9a–d). However, this picture changed when normalizing these results to the total number of genes per class (Figure 1c), which rendered ion channels, enzymes and nuclear receptors to be more likely, in relative terms, to contribute DHETGs (Figure 9a). This share also differed by cell type to a surprising degree; while ion channel genes consistently dominated in relative target numbers in most cells, in line with their essential role in determining the electrophysiological characteristics of each neuron type, others, like nuclear receptors (dominating in murine L4/5 IT and Pvalb neurons) or kinases (dominating in L2/3 cells), depended on cell type.

To investigate if this class specificity also extends to druggable targets as a whole compared to the remaining genome, we conducted an exemplary intra-regional analysis for two representative cell types, L2/3 and Pvalb neurons of murine ACA using all genes available in the CytosploreViewer output (*N* ≈ 45,768). In L2/3, the non-conservative genome-wide analysis yielded 156 DHETGs (156/45,768 = 0.341%) compared to 31 hits within the IDG druggable subset (31/7254 = 0.427%); for the conservative analysis, the corresponding numbers were 3/45,768 (0.0066%) and 1/7254 (0.0138%). In Pvalb interneurons, the non-conservative genome-wide analysis yielded 282 DHETGs (18/45,768 = 0.616%) compared to 46 DHETGs within the IDG subset (46/7254 = 0.634%); for the corresponding conservative analysis, the numbers were 18/45,768 (0.0393%) and 8/7254 (0.1103%). Thus, in both examples, DHETG hit rates were somewhat higher within the druggable subset than genome-wide. Importantly, both analyses indicate that highly selective genes are rare overall under stringent criteria, suggesting that limited selectivity is not exclusive to druggable targets.

## 4. Discussion

### 4.1. Lack of Differential Gene Expression Poses a Principal Biological Limit to Neuron Type-Specific Pharmacology

We here investigated the generic possibility to translate cellular targets, identified by circuit neuroscience experiments as relevant for psychiatric disorders, into molecular druggable targets, focusing on three highly translationally relevant brain areas in mice (ACA, mPFC and hippocampus) and the ACA in humans. Even though over 7200 putatively druggable targets (IDG list) were investigated and only a relatively limited number of comparisons across cortical areas and cell types were conducted, none of the target cell types showed fully selective expression of any target gene in the murine or human neocortex, and only very few emerged in the hippocampus. Region-specific targets were especially scarce. It is fair to assume that even the latter would not hold up in a larger number of comparisons against subcortical types of neurons or other cell types of the brain and body. Therefore, it is highly unlikely that selectively expressed neuronal targets even *exist*, at least in cortical neurons.

This finding implicates that the low selectivity of gene expression constitutes a principal biological limit to the translation of cellular into molecular targets in psychiatry. Even if causality between very specific cell types and symptoms can be established, such cell types can most likely not be addressed with the intended pharmacological specificity.

### 4.2. Selecting Suitable Targets with Limited Specificity Based on Biologically Informed Contrasts

Our results imply that only *relative* specificity of expression can be assumed for future targets to be developed. Our less conservative (“one-vs-all”) target selection approach, balancing sensitivity and selectivity, points towards that possibility, capturing genes consistently enriched in the target population compared to the rest. This approach allows for the detection of genes that are expressed more strongly in the target cell type relative to the group average, even if they are not consistently elevated compared to every individual subtype.

The stepwise intersection in both inter- and intra-regional comparisons also reflects that the reality of the limited selectivity of gene expression requires one to make biologically informed choices about which contrast of cell types is physiologically or pharmacologically more relevant than others. For example, when targeting L2/3 pyramidal cells, it is likely less relevant if the same molecular target is expressed in L5 pyramidal cells (which are excited by L2/3 neurons anyway [39]) than in any type of interneuron (which may inhibit them); when targeting ACA, it is likely more relevant to leave sensory and motor areas unperturbed than mPFC, which shares many cognitive functions and pathological aberrations with ACA [40]; and when targeting Pvalb interneurons, it is more important to avoid VIP interneurons (which inhibit Pvalb cells [41]) than Sst neurons.

Our analysis also showed some general rules that could guide such a search for targets expressed with relative specificity. For example, to modulate activity in a certain cortical area, targeting pyramidal cells, especially L2/3 neurons, might allow for greater specificity than targeting interneurons, given the identified lack of globally consistent transcriptional divergence across regions in interneurons. However, when it is rather critical to modulate certain interneurons, such as Pvalb neurons [12,42], then at least some specificity can be achieved when aiming to leave the hippocampus unperturbed, given that neocortical interneurons showed some transcriptional divergence from their hippocampal counterparts (Figure 2 and Figure 5).

### 4.3. Psychiatric Relevance of Genes Expressed with Moderate Selectivity

In that regard, our results also serve as a database to start from for future target development, which seeks to modulate mPFC, ACA or the hippocampus using molecular targets with the highest biologically possible selectivity. To support such future endeavours, we aggregated the human → mouse translated IDG target list (Supplementary File S3), usable cell type lists (Supplementary Files S1 and S2) and all detailed results for every individual comparison (Supplementary Files S4–S8). As preliminary exploration of the future potential of such results, we conducted a literature search for the target genes that were expressed with relatively high selectivity (intersection of at least two inter-regional comparisons and outcome of the conservative intra-regional analysis) and compiled the result in Table 4 for the murine neocortex.

Several further targets occurring with lower selectivity in neocortical excitatory cells have also been implicated in psychiatric disorders and are mostly addressable with existing compounds:

- *Htr2c*: downregulated in schizophrenia patients [56];
- *Gabra5*: NAM produces rapid and sustained antidepressant responses [57];
- *Kcns3*: role in calcium-dependent regulation of excitability and microglial inflammatory responses [45];
- *Npyr2*: KO reduces anxiety and stress response [58];
- *Oprk1*: antagonists are in clinical development for depression [59];
- *Trpc5*: inhibition induces antidepressant and anxiolytic effects in rodents [60].

Also, interneurons contained further DHETGs at lower selectivity:

- *Cnr1*: polymorphism (rs1049353) has been associated with antidepressant treatment resistance [61];
- *Kcnd2*: associated with autism susceptibility [62];
- *Kcnj3*: genetic association with schizophrenia [63];
- *Trpc6*: target for fast-acting antidepressants [64].

We did not find psychiatric associations for the three DHETGs resulting from the conservative intra-regional comparisons in mPFC, but several hits from the same analysis in ACA did have such links, such as *Kcnc1*, *Kcnh5*, *Grm1* and *Grin3a* (see above).

In the conservative intra-regional hippocampus analysis, two DHETGs were found: CA2 neurons showed higher expression of *Gpr12*, which is implicated in neurodevelopment and neurite outgrowth, but its physiological role remains poorly defined, and pharmacological tools are limited [65]. In Pvalb neurons, *Kcnc1* was identified just like in their neocortical counterparts (Table 4).

The human inter-regional analysis also revealed several genes with prior associations to psychiatric disorders. However, these generally showed only weak selectivity, occurring in only one contrast (mostly against sensory cortex; Figure 6a) and include the following: *GRIA1* (genetic and histological associations with schizophrenia [66]), *GRIK1* (Table 4), *GABRG1* (genetically associated with alcohol dependence [67], *ASIC2* in VIP neurons (deletion reduces fear- and panic-like responses in rodents [68]), and *KCND2* (associated with autism [62]). In the intra-regional human analysis, only two DHETGs emerged, both in Sst neurons and both not yet targetable pharmacologically. While we found no association between *FLT3* and psychiatric diseases, *SATB1* mutations in humans lead to neurodevelopmental abnormalities, which are recapitulated by its knockout in mice [69].

Additionally, we used a recently published database of targets of clinically used psychiatric and neurological compounds [70], converted its protein entries into human and mouse gene names and matched the resulting list against the DHETG lists resulting from the least conservative intra-regional DGE analyses in mouse ACA/mPFC (combined), mouse hippocampus and human ACA (Supplementary Table S2). We found that in each case, only 4.3–4.6% of the identified DHETGs correspond to clinically used targets, highlighting a considerable potential for future target discovery.

### 4.4. Limitations and Future Directions

Our study aimed to make maximal use of the data contained in two of the largest single-cell transcriptomic databases in existence: the AI Mouse SmartSeq database containing 76,307 transcriptomes across 20 areas of the neocortex and hippocampus, and the AI Human SmartSeq database containing 49,417 transcriptomes of six areas, for examining the translationally most relevant cell types regarding the maximum number of putatively druggable targets (IDG list of the Pharos database). Nevertheless, equally relevant comparisons with the many psychiatrically implicated subcortical areas in the basal ganglia, thalamus, and midbrain as well as with cell types of vital organs of the body could not be included due to obvious logistical limitations of handling the combinatorial “explosion” of the number of contrasts and difficulties in comparing across datasets. Still, the present analysis was sufficient to answer the core research question regarding the principal biological limit of expression specificity in cortical cell types. In future, the identified targets with moderate specificity need to be checked carefully against further relevant cell types. Additionally, our analysis motivates a similar study for subcortical areas: the laminar structure of the neo-, paleo- and archicortex might be particularly ill-suited for the search for selectively expressed drug targets because it is built with the same “building blocks” or “circuit elements” of excitatory and inhibitory cell types all over the cortical mantle [71], which could at least explain the low number of DHETGs in the inter-regional comparison. Subcortical brain regions that are more unique in architecture may yield a higher number of DHETGs and may, therefore, be a better access point for treatment of psychiatric disorders that also involve these structures. However, our results from the intra-regional analysis showing that even contrasts between very different types of neurons yield only rather limited numbers of DHETGs (typically between 50 and 300 out of 7254, Figure 3) should raise caution regarding hopes for different results in the analysis of such regions.

Furthermore, the numerical conditions of our analysis need to be highlighted, especially regarding the fold-expression change used as a criterion: we set a three-fold difference in expression level (1.585 in terms of difference of log2-transformed CPM values) as a criterion for selective expression (as long as the Bonferroni-corrected *p* < 0.05 was also fulfilled). This value was chosen in alignment with our previous study, where it yielded a reasonable amount of GPCR targets [11]; it represents a pragmatic definition, balancing the risk of inflated candidate lists at more permissive and biologically less relevant cutoffs (e.g., 2-fold) against an excessively stringent criterion, leaving almost no results despite biologically appreciable differences in gene expression (e.g., five-fold). As an exemplary sensitivity analysis of this choice, we investigated the differences between a two-, three- and five-fold threshold for two important cell types in the mouse ACA intra-regional comparisons. As expected, DHETG counts decreased monotonically with increasing stringency, yielding the following numbers at the intersection of all eight comparisons: L2/3: 2-fold = 2-, 3-fold = 1-, 5-fold = 0; and PV: 2-fold = 14-, 3-fold = 8-, 5-fold = 4.

In contrast to our previous analysis of only one cell type, we did not take the absolute expression levels in the contrast set (GE_Set-C_) nor the share of cells with any expression (beta) into account [11] for the analysis of the mouse dataset. When including such constraints in the analysis of the human data, alongside a relaxation of the intersectional strategy and of the *p*-value correction (FDR), it became apparent that the usage of a standard beta threshold (>20%) can sharpen the biologically plausible DHETG selection without excluding too many potential targets expressed with relative specificity. In contrast, the demand of a virtual lack of expression in the contrast cells appeared to be a drastic constraint eliminating the vast majority of targets. This example highlights that the already low number of DHETGs would become even lower when imposing more conservative numeric constraints such as demanding a higher fold-change or thresholding beta or GE_Set-C_. Therefore, our approach captures genes that are, on average, more highly expressed in the target group but not necessarily specific to it. While the differences are likely functionally meaningful, particularly if they are shared across related neuronal populations, they should not be interpreted as strict cell type markers.

However, relaxing the comparison from a strict intersectional to a class-based strategy also gives rise to the phenomenon that genes whose expression level varies across large scales can still be highlighted as significantly expressed in multiple cells. This is illustrated by *Gstm1*, which appears in the intersection of all neocortical areas and the non-conservative intra-cortical comparison for both L2/3 and L6 pyramidal neurons of the mPFC, and is also still expressed in L6 IT of ACA (higher compared to sensory cortex). This is possible because *Gstm1* expression is more than three-times higher in mPFC L6 IT (log_2_(CPM) = 7.8) than in mPFC L2/3 and ACA L6 IT (both log_2_(CPM) = 5.4), in which the expression is again more than three-times higher than in the other cell groups (e.g., sensory cortex L6 IT log_2_(CPM) = 2.9; sensory cortex L2/3 log_2_(CPM) = 2.0, motor cortex L2/3 log_2_(CPM) = 1.7; Supplementary Files S4 and S5). Surveying all results from the non-conservative intra-regional analysis of mouse mPFC and ACA, we found that *Gstm1* is not an exception but that out of the 303 DHETGs identified across all analysed ACA cell types, 43 were duplicates or triplicates (14.2%), whereby four DHETGs were shared between three cell types and a further 31 by two cell types. In mPFC, out of 190 DHETG hits, 12 (6.3%) were shared by two cell types and none by more than two (Supplementary Table S3). Similarly, when replacing the intersectional approach altogether in favour of a *majority-rule* (i.e., a gene has to be a DHETG in 5 out of 8 pairwise intra-regional comparisons), as an alternative to relax constraints, this phenomenon was even more common. While the number of DHETGs identified in the eight target cell types of murine ACA in the intra-regional comparison (320) was similar to our standard non-conservative approach (303), 93 (29%) such hits appeared in more than one cell type: 68 DHETGs appeared in 2, 23 in 3, and 2 in 4 cell types, respectively (Supplementary Figure S1). In addition to the problem that a majority rule treats all cell type contrasts as equally important, not aligned with the biological reality of neural circuits, this result of a high number of shared DHETGs argues against its use for target discovery.

A principal limitation of our study, and DGE analysis in general, is that fold-change differences in mRNA expression and, by extension, most numeric constraints imposed on the transcriptional data may not translate into actual differences in expression and cellular function of the resulting proteins. Post-transcriptional and post-translational mechanisms as well as the dynamics of amplification of intra-cellular signalling, and of sequestration, compartmentalization and degradation of proteins may skew the relationship between relative mRNA expression levels captured by fold-change, its *p*-value and beta as well as ultimate protein function. For example, differences in amplification in a signalling cascade, as mediated by differences in intracellular localization or modulating factors, could theoretically compensate for differences in mRNA expression levels or, in reverse, cause substantial differences between two cell types, even if they show similar expression levels. Our exemplary human ACA analysis showed, however, that the alternative of imposing absolute absence of expression in the contrast cell type as criterion to circumvent this problem would leave hardly any targets.

Therefore, DGE differences can only be one out of several perspectives to consider when aiming for addressing neuron types selectively. Furthermore, the DHETGs identified here and in similar investigations need to be verified at the protein level, before drawing strong conclusions regarding a differentially expressed target. Nevertheless, this study illustrates the considerable challenges ahead, when aiming to translate cellular targets identified with circuit neuroscience techniques into molecular targets and encourages us to take on this endeavour with greater effort, using criteria for moderate selectivity, as its success ultimately defines the translational value of circuit-neuroscience studies.

## 5. Conclusions

We identified the lack of high selectivity of the expression of genes encoding druggable targets as a principal biological limit for specific pharmacological manipulation of therapeutically relevant types of cortical neurons. Nevertheless, our large-scale analysis represents a starting point when approaching the endeavour of neuropharmacological target discovery when prioritizing the selectivity of therapeutic interventions and it discovered several candidates worth of further exploration. Identifying genes that are differentially expressed in specific psychiatrically implicated populations of neurons may enable the development of more targeted therapeutic strategies, with the potential for improved efficacy and reduced side effects in the treatment of psychiatric diseases.

## Figures and Tables

**Table 1:** Selection of cell types for analysis of murine ACA and mPFC. L refers to the neocortical layer and denotes exclusively excitatory neurons from that layer; the remaining cells are inhibitory interneurons. ^A^ only exists in ACA, not mPFC. * used only for intra-regional comparison, due to expected high similarity of L4IT, L4/5 IT and L5IT subtypes and unclear relevance of L6 CT, respectively. ** used only for inter-regional comparison, to ensure similarity of cell type across regions, which would be uncertain for L4-IT and L4/5-IT due to differential expansion and function of L4 in sensory vs. frontal areas. *** used only as contrast set.

Cell Type	Subclass Label in Metadata
L2–3	L2–3 IT Otof, L2–3 IT Cxcl14 (nur ACA)
L4–5 IT *	L4 IT, L4/5 IT, L5 IT, RSP/ACA L4/5 IT ^A^, RSP/ACA L4/5 IT Scnn1a ^A^
L5 IT **	L5 IT
L5 ET	L5 ET
L6 IT	L6 IT
L6 CT *	L6 CT
L6b ***	L6b
Pvalb	Pvalb
Sst	Sst, Sst Chodl
VIP	Vip
All glutamatergic	IT RHP Dcn ^A^, L2/3 IT Cxcl14 ^A^, L2/3 IT Otof, L4 IT, L4/5 IT, L5 ET, L5 IT, L6 CT, L6 IT, L6b, NP, RSP/ACA L4/5 IT ^A^, RSP_ACA IT Scnn1a ^A^
All GABAergic	Lamp5, Lamp5 Lhx6, Meis2, Serpinf1, Sncg, Sncg/Ndnf HPF, Sst, Sst Chodl, Vip, Pvalb

**Table 2:** Selection of cell types for analysis of murine hippocampus. CA1/2/3, DG and SUB refer to excitatory cells, the remainder to inhibitory interneurons. * only used as contrast set.

Cell Type	Subclass Label in Metadata
CA1	CA1sp, CA1sp/SUB-sp Kcnip1
CA2	CA2sp/IG
CA3	CA3sp
DG	DG
SUB	SUB-Sp Ndst4, CA1sp/SUB-sp Kcnip1
Pvalb	Pvalb
Sst	Sst
Vip	Vip
Sncg + Lamp5 *	Lamp5, Lamp5 Lhx6, Sncg, Sncg/Ndnf HPF

**Table 3:** Selection of cell types for analysis of human ACA (corresponding to the area label CgC, cingulate cortex, in the original dataset). * only used as contrast set. ** also contains L2/3 pyramidal neurons.

Cell Type	Subclass Label in Metadata
L5 ET *	L5 ET
L5/6 NP *	L5/6 NP
L6 CT *	L6 CT
L6b *	L6b
IT *	IT **, L4 IT, L5/6 IT Car3
PVALB	PVALB
SST	SST
VIP	VIP
PAX6 *	PAX6
LAMP5 *	LAMP5

**Table 4:** DHETGs with psychiatric relevance resulting from intra- and inter-regional analysis of murine ACA and mPFC (intersection of at least 2 inter-regional comparisons).

Gene	Region	Neuron	Link to Psychiatric Symptoms	References
*Kcnab1*	*ACA*	L2/3p	downregulated by several antipsychotics	[43]
*Kccn3*	*mPFC*	L2/3p	known pharmacological activators (CyPPA) and blockers (apamin) downregulation may improve cognition in schizophrenia	[44,45]
*Grm8*	*ACA*	L2/3p	regulation of limbic motivation and stress circuits mGluR8 agonists/PAMs show anxiolytic, antiepileptic, and anti-addictive effects	[46]
*Gpr83*	*ACA*	L2/3p	anxiolytic effects	[47]
*Grin3a*	*mPFC*	L5 IT, L6 IT, Sst	associated with schizophrenia-related phenotype	[48]
*Kcnh5*	*ACA*	L4/5 IT	deletion induces autism phenotype	[49]
*Htr4*	*ACA*	L5 ET	pro-cognitive effect of 5-HT4R agonist in humans	[50]
*Slc6a7*	*ACA*	L5 ET	Proline-transporter affecting glutamatergic signalling and behaviour in rodents	[51]
*Ntsr2*	*mPFC*	L6 IT	implicated in stress responses	[52]
*Fxyd6*	*mPFC*	Sst, Pvalb, VIP	regulates Na/K-ATPase activity and neural excitability: shows developmental and genetic alterations associated with early-onset schizophrenia	[53]
*Grm1*	*ACA*, *mPFC*	Sst	implicated in schizophrenia and explored as drug target	[54]
*Kcnc1*	*ACA*, *mPFC, Hip*	Pvalb	Crucial potassium channel (Kv3) for fast-spiking behaviour; explored as target for cognitive and psychotic symptoms	[55]

## Data Availability

All supporting data are contained as Supplementary Files S1–S8 of this publication. Original expression datasets can be found on the website of the Allen Institute for Brain Science: https://celltypes.brain-map.org/ (accessed on 21 September 2023)).

## Supplementary Materials

The following supporting information can be downloaded at: https://www.mdpi.com/article/10.3390/biomedicines14040823/s1, Supplementary File S1: Mouse cell type sets; Supplementary File S2: Human cell type sets; Supplementary File S3: target genes; Supplementary File S4: murine ACA DHETG results; Supplementary File S5: murine mPFC DHETG results; Supplementary File S6: murine hippocampus DHETG results; Supplementary File S7: Human ACA DHETG results with conservative analysis. Supplementary File S8: Human ACA DHETG results with non-conservative analysis. Supplementary Figure S1: Majority rule sensitivity analysis of conservative intra-regional differential gene expression in murine ACA. Supplementary Table S1: Expression of established marker genes across murine annotated ACA cell types. Supplementary Table S2: Overlap between identified DHETGs and targets of approved neuropsychiatric drugs across brain regions. Supplementary Table S3: DHETGs recurring across multiple target cell type-specific DHETG lists in the non-conservative intra-regional analyses of murine ACA and mPFC.

## Abbreviations

The following abbreviations are used in this manuscript:

ADHD	Attention-deficit–hyperactivity disorder
ACC	Anterior cingulate cortex
ACA	Anterior cingulate area, equivalent to ACC
mPFC	Medial prefrontal cortex, comprising PL and IL areas
DG	Dentate gyrus
CA	Cornua ammonis
Sub	Subiculum
MTG	Medial temporal gyrus
CgC	Cingulate Cortex
DGE	Differential gene expression
DHETGs	Differentially higher expressed target gene
Se	Sensory areas
Mo	Motor areas
