# Supplementary material for: Comprehensive Assessment of Druggable Targets in Cortical Neurons Reveals Biological Limits of Cell Type-Specific Neuropharmacology
Source: Biomedicines. 2026 Apr 3;14(4):823. doi: 10.3390/biomedicines14040823 (PMC13112967; doi:10.3390/biomedicines14040823)
Supplement: Supplementary file 1 [file biomedicines-14-00823-s001.zip › Ripp & Kätzel Supplementary Information.pdf]

Supplementary Information

Comprehensive assessment of druggable targets in cortical neurons reveals biological limits of cell type-specific neuropharmacology

Leonie Ripp[1] and Dennis Kätzel [1,*]

1 Institute of Applied Physiology, Ulm University
* Correspondence: dennis.kaetzel@uni-ulm.de

Supplementary Figures

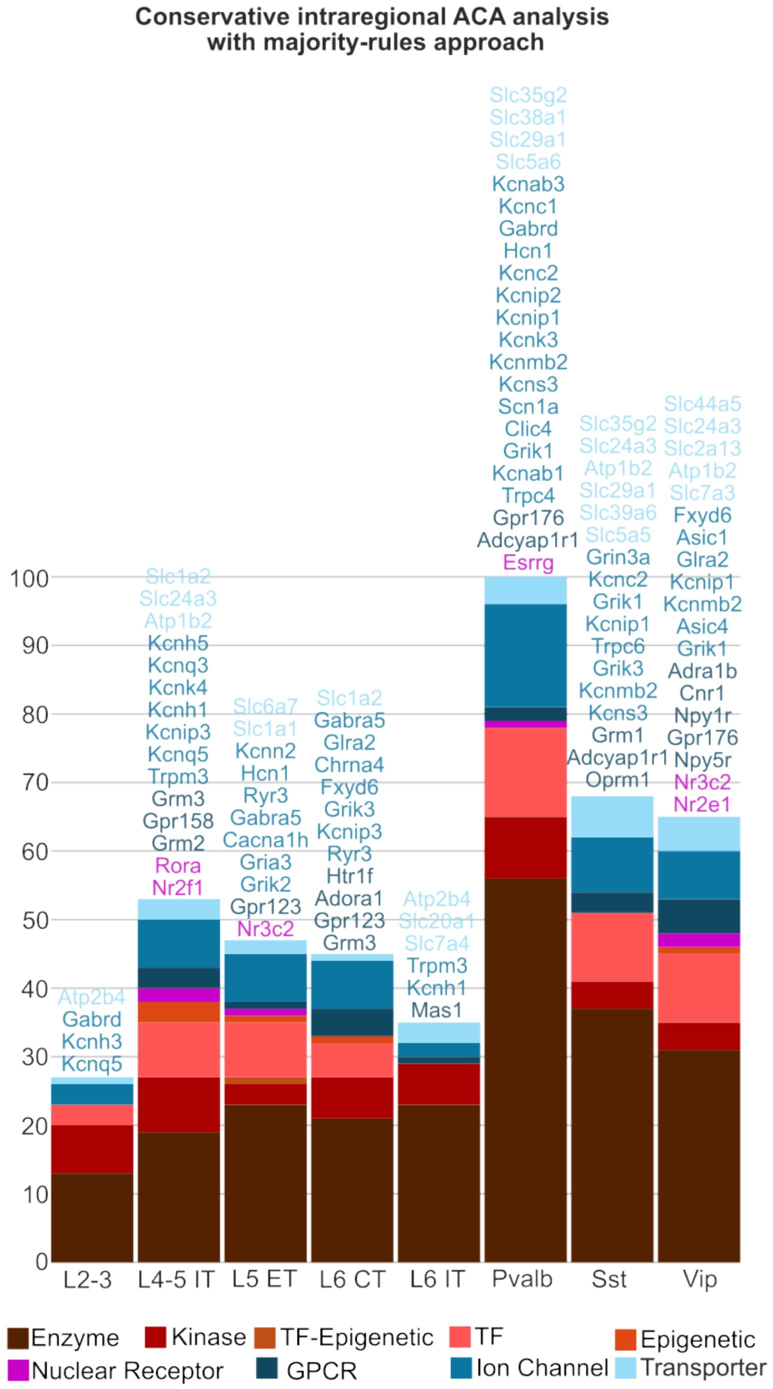

Supplementary Figure S1 | Majority-rule sensitivity analysis of conservative intra-regional differential gene expression in murine ACA. The number of DHETGs identified for each ACA cell type when applying a majority-rule criterion instead of complete intersection is shown. For each target cell type, a gene was required to be a DHETG in at least 5 of the 8 pairwise contrasts against the remaining ACA cell types (≥5/8 rule). Genes are colour-coded by target class, as in main Figure 3.

Supplementary Tables

Marker gene	Target cell type	Contrast set cell types	(Range of) Diff_Mean
VIP	VIP	L2-3, L4-5 IT, L5 ET, L6 IT, L6CT, L6b, Pvalb, Sst	13.87-14.41
Pvalb	Pvalb	L2-3, L4-5 IT, L5 ET, L6 IT, L6CT, L6b, Sst, VIP	7.84-9.07
Sst	Sst	L2-3, L4-5 IT, L5 ET, L6 IT, L6CT, L6b, Pvalb, VIP	12.87-13.49
Gad1	VIP	All glutamatergic cell types combined	10.43
Gad1	Pvalb	All glutamatergic cell types combined	10.57
Gad1	Sst	All glutamatergic cell types combined	9.67
Gad2	VIP	All glutamatergic cell types combined	8.42
Gad2	Pvalb	All glutamatergic cell types combined	8.61
Gad2	Sst	All glutamatergic cell types combined	9.12
Slc17a7	L2-3	All GABAergic cell types combined	8.25
Slc17a7	L4-5 IT	All GABAergic cell types combined	8.43
Slc17a7	L5 ET	All GABAergic cell types combined	8.34
Slc17a7	L6 IT	All GABAergic cell types combined	9.00
Slc17a7	L6 CT	All GABAergic cell types combined	8.91

Supplementary Table S1 | Expression of established marker genes across murine annotated ACA cell types. Established marker genes for major inhibitory subclasses and excitatory neurons were examined across the annotated ACA cell types. VIP, Pvalb, and Sst showed strong enrichment in their respective interneuron subclasses across all pairwise contrast sets. Likewise, the inhibitory markers *Gad1* and *Gad2* were strongly enriched in VIP, Pvalb, and Sst interneurons relative to all glutamatergic cell types combined, whereas the excitatory marker *Slc17a7* encoding vGlut1, showed strong enrichment in excitatory cell types relative to all GABAergic cell types combined. These results demonstrate that the annotated cell-types are consistent with established marker gene expression patterns. For reference, a 3-fold and 10-fold increase of expression in the target compared to the contrast cell-type corresponds to a Diff_Mean value of 1.585 and 3.322, respectively.

**Regions**	**Number of identified DHETGs**	**DHETGs targeted by approved drugs (%)**	**DHETGs targeted by approved drugs**
**Mouse ACA + mPFC**	*474*	*4.43*	*Ache (Enzyme), Adora1 (GPCR), Adra1b (GPCR), Adra1d (GPCR), Cacna1g (Ion Channel), Chrm3 (GPCR), Flt3 (Kinase), Gabra2 (Ion Channel), Gabra5 (Ion Channel), Gabrd (Ion Channel), Gabrg3 (Ion Channel), Grik1 (Ion Channel), Hmgcr (Enzyme), Htr2c (GPCR), Kcna1 (Ion Channel), Kcnq3 (Ion Channel), Maob (Enzyme), Mgll (Enzyme), Nr3c2 (Nuclear Receptor), Oprk1 (GPCR), Oprm1 (GPCR)*
**Mouse HIP**	*70*	*4.29*	*Adra1b (GPCR), Chrna4 (Ion Channel), Gabrd (Ion Channel)*
**Human ACC**	*175*	*4.57*	*ADRA1B (GPCR), CHRNA7 (Ion Channel), FLT3 (Kinase), GABRA2 (Ion Channel), GABRD (Ion Channel), GRIK1 (Ion Channel), HTR2C (GPCR), PDE3A (Enzyme)*

Supplementary Table S2 | Overlap between identified DHETGs and targets of approved neuropsychiatric drugs across brain regions. Shown are the total number of DHETGs for each region, the proportion targeted by approved drugs, and the corresponding genes. For mouse ACA + mPFC, all identified DHETGs of the non-conseravtive analysis were included. In mouse hippocampus, only DHETGs identified in the complete intersection of the less-conservative analysis were considered, and for human ACC, only DHETGs identified in the Benjamini-Hochberg-corrected analysis were included.

Region	Cell types in whose DHETG lists the gene occurs	Shared DHETG(s)
**ACA**	*L2-3, L4-5 IT*	*Camk1 (Enzyme), Pdp1 (Enzyme), Pdzrn3 (Enzyme), Cacng3* (Ion Channel), Kcnq5* (Ion Channel), Epha4 (Kinase), Epha7 (Kinase), Etnk2 (Kinase), Mapk4 (Kinase)*
	L2-3, L5 ET	*Dgkb* (Enzyme), Epha6* (Kinase), Bhlhe40 (TF)*
	L2-3, L6 IT	*Arl15 (Enzyme), Dgkb* (Enzyme), Rasl10a (Enzyme), Cacng3* (Ion Channel), Kcnq5* (Ion Channel), Camkk1 (Kinase), Epha6* (Kinase)*
	L4-5, L5 ET	*Nlgn1 (Enzyme)*
	L4-5 + L6 IT	*Ptprk (Enzyme), Cacng3* (Ion Channel), Kcnq5* (Ion Channel), Trpm3 (Ion Channel), Etv6 (TF)*
	L4-5, Pvalb	*Rora (Nuclear Receptor)*
	L4-5, Sst	*Cacna2d3 (Ion Channel)*
	L4-5, VIP	*Kcnt2 (Ion Channel)*
	L5 ET, L6 CT	*Crym (Enzyme), Gpr123 (GPCR), Ryr3 (Ion Channel), Prkcg (Kinase), Bcl11b (TF)*
	L5 ET, L6 IT	*Dgkb* (Enzyme), St6galnac5 (Enzyme), Epha6* (Kinase)*
	L5 ET, Pvalb	*Ndst3 (Enzyme), Hcn1 (Ion Channel)*
	L6 CT, L6 IT	*B3galt2 (Enzyme), Sulf1 (Enzyme)*
	L6 CT, VIP	*Fxyd6 (Ion Channel)*
	L6 IT, Sst	*Grm1 (GPCR)*
mPFC	*L2-3, L4-5 IT*	*Epha4 (Kinase)*
	L2-3, L6 IT	*Arl15 (Enzyme), Atp2b4 (Transporter), Dgkb (Enzyme), Gstm1 (Enzyme), Kcnq5 (Ion Channel), Rasl10a (Enzyme)*
	L5 ET, L6 CT	*Crym (Enzyme), Nfia (TF), Nos1ap (Enzyme)*
	L5 ET, Pvalb	*Hcn1 (Ion Channel)*
	Pvalb, Vip	*Erbb4 (Kinase)*

Supplementary Table S3 | DHETGs recurring across multiple target cell-type-specific DHETG lists in the non-conservative intra-regional analyses of murine ACA and mPFC. Genes that were identified as DHETGs in more than one target cell-type are stated in column 3 alongside the two cell-types in which they occurred (column 2). (The second column does not denote a direct comparison between the listed cell types.) Asterisks (*) indicate genes that occurred in three target cell-type-specific DHETG lists
